# Supplementary material for: Creation of a 3D Goethite–Spongin Composite Using an Extreme Biomimetics Approach
Source: Biomimetics (Basel). 2023 Nov 9;8(7):533. doi: 10.3390/biomimetics8070533 (PMC10668986; doi:10.3390/biomimetics8070533)
Supplement: Supplementary file 1 [file biomimetics-08-00533-s001.zip › biomimetics-2685497-supplementary.pdf]

biomimetics

MDPI

Supplementary information for article

Creation of a 3D Goethite–Spongin Composite Using an Extreme Biomimetics Approach

Anita Kubiak [1,2,*], Alona Voronkina [3,4], Martyna Pajewska-Szmyt [2], Martyna Kotula [1,2], Bartosz Leśniewski [1,2], Alexander Ereskovsky [5], Korbinian Heimler [6], Anika Rogoll [6], Carla Vogt [6], Parvaneh Rahimi [3], Sedigheh Falahi [3], Roberta Galli [7], Enrico Langer [8], Maik Förste [9], Alexandros Charitos [9], Yvonne Joseph [3], Hermann Ehrlich [2,10] and Teofil Jesionowski [10,*]

[1] Faculty of Chemistry, Adam Mickiewicz University, Uniwersytetu Poznanskiego 8, 61-614 Poznan, Poland; markot6@amu.edu.pl (M.K.); barles5@amu.edu.pl (B.L.)
[2] Center of Advanced Technology, Adam Mickiewicz University, Uniwersytetu Poznanskiego 10, 61-614 Poznan, Poland; mpszmyt@amu.edu.pl (M.P.-S.); herehr@amu.edu.pl (H.E.)
[3] Institute of Electronics and Sensor Materials, TU Bergakademie Freiberg, Gustav-Zeuner-Str. 3, 09599 Freiberg, Germany; voronkina@vnmu.edu.ua (A.V.); parvaneh.rahimi@esm.tu-freiberg.de (P.R.); sedigheh.falahi@doktorand.tu-freiberg.de (S.F.); yvonne.joseph@esm.tu-freiberg.de (Y.J.)
[4] Department of Pharmacy, National Pirogov Memorial Medical University, Vinnytsya, Pyrogov Street. 56, 21018 Vinnytsia, Ukraine
[5] IMBE, CNRS, IRD, Aix Marseille University, Station Marine d'Endoume, Rue de la Batterie des Lions, 13007 Marseille, France; alexander.ereskovsky@imbe.fr
[6] Institute of Analytical Chemistry, TU Bergakademie Freiberg, Leipziger Str. 29, 09599 Freiberg, Germany; korbinian.heimler@chemie.tu-freiberg.de (K.H.); anika.rogoll@chemie.tu-freiberg.de (A.R.); carla.vogt@chemie.tu-freiberg.de (C.V.)
[7] Department of Medical Physics and Biomedical Engineering, Faculty of Medicine Carl Gustav Carus, TU Dresden, Fetscherstr. 74, 01307 Dresden, Germany; roberta.galli@tu-dresden.de
[8] Institute of Semiconductors and Microsystems, TU Dresden, Nöthnitzer Str. 64, 01187 Dresden, Germany
[9] Institute for Nonferrous Metallurgy and Purest Materials (INEMET), TU Bergakademie Freiberg, Leipziger Str. 34, 09599 Freiberg, Germany; maik.foerste@inemet.tu-freiberg.de (M.F.); alexandros.charitos@inemet.tu-freiberg.de (A.C.)
[10] Faculty of Chemical Technology, Institute of Chemical Technology and Engineering, Poznan University of Technology, Berdychowo 4, 60-965 Poznan, Poland
* Correspondence: anikub@amu.edu.pl (A.K.); teofil.jesionowski@put.poznan.pl (T.J.)

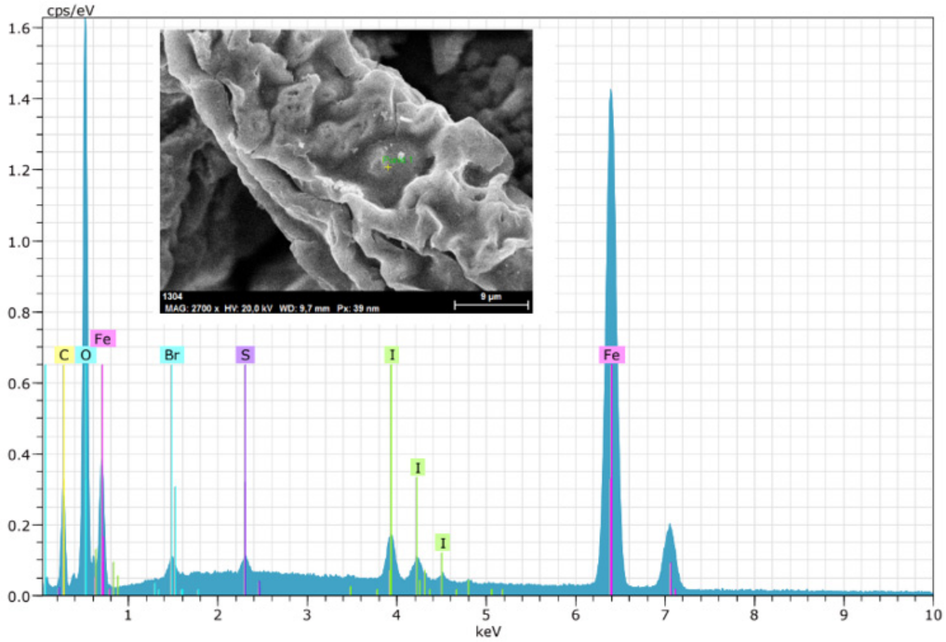

El	OZ	Serie	Netto	norm. C [Gew.%]	Atom. C [At.%]	Fehler (1 Sigma) [Gew.%]
O	8	K-Serie	53444	27,7	46,4	2,93
Fe	26	K-Serie	145730	54,3	26,0	1,31
C	6	K-Serie	8937	11,6	25,9	1,50
I	53	L-Serie	17649	4,7	1,0	0,15
Br	35	L-Serie	3414	1,3	0,4	0,09
S	16	K-Serie	2709	0,4	0,3	0,04
			Summe:	100,0	100,0	

Figure S1. EDX measurements (1) of FeISpongin composite after ultrasound treatment.

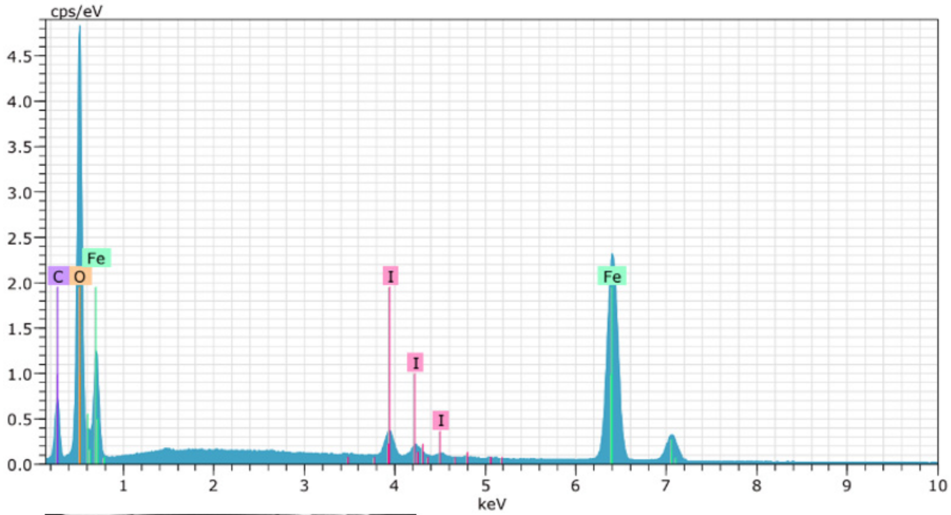

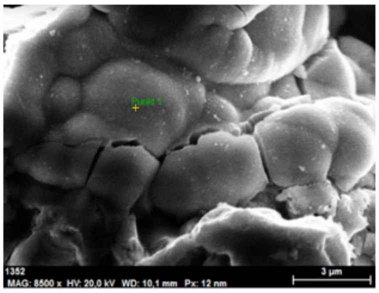

El	OZ	Serie	Netto	norm. C [Gew.%]	Atom. C [At.%]	Fehler (1 Sigma) [Gew.%]
O	8	K-Serie	164048	39,7	56,9	3,65
C	6	K-Serie	22860	12,9	24,7	1,38
Fe	26	K-Serie	223886	42,6	17,5	0,96
I	53	L-Serie	36803	4,8	0,9	0,14
			Summe:	100,0	100,0	

Figure S2. EDX measurements (2) of FeISpongin composite after ultrasound treatment
