# Supplementary figures and images for: Creation of a 3D Goethite–Spongin Composite Using an Extreme Biomimetics Approach
Source: Biomimetics (Basel). 2023 Nov 9;8(7):533. doi: 10.3390/biomimetics8070533 (PMC10668986; doi:10.3390/biomimetics8070533)

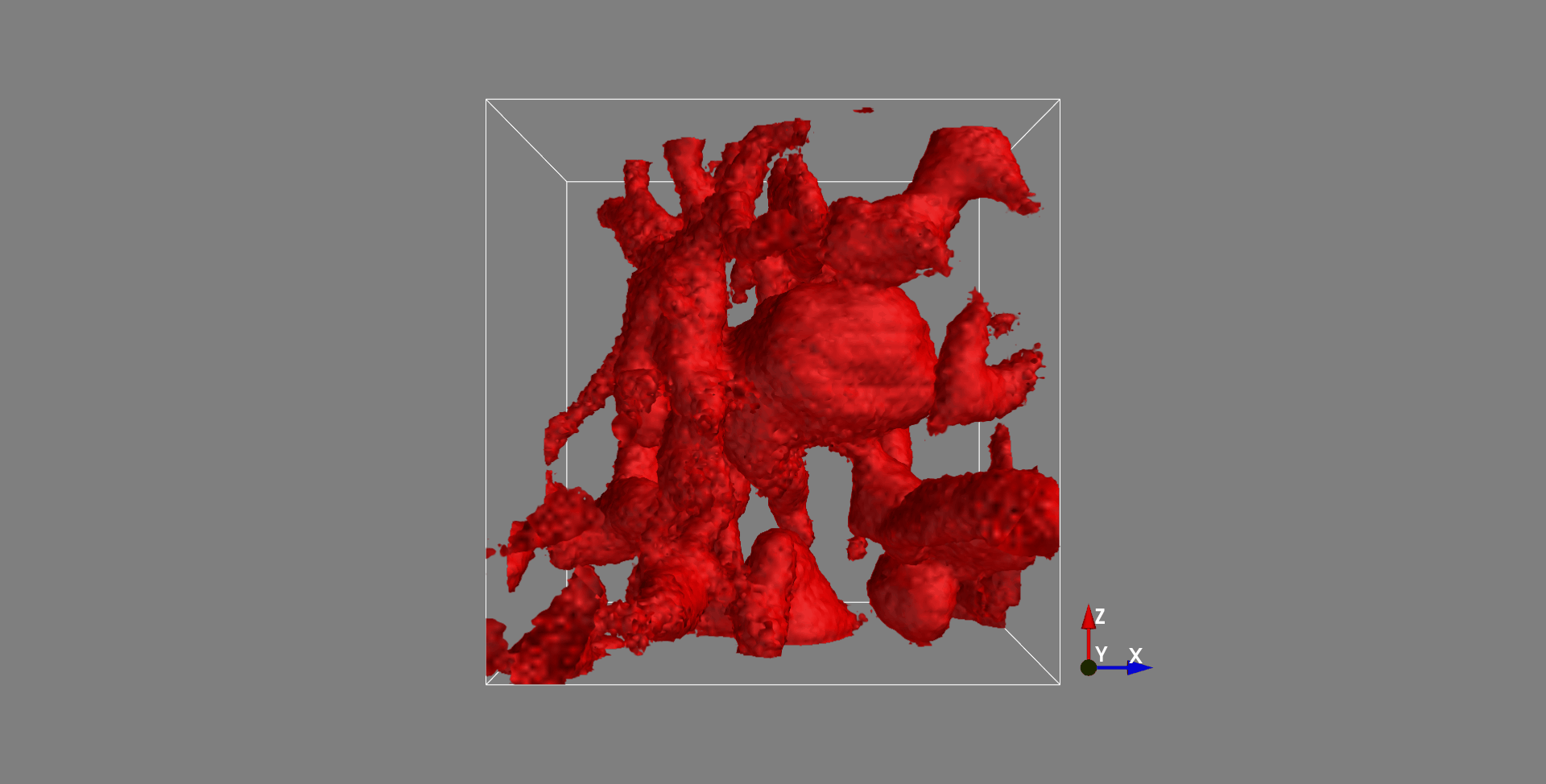

Supplement: Supplementary file 1 [file biomimetics-08-00533-s001.zip › Video S1.gif]
